# Kidney transplant access for children and young adults with congenital anomalies of the kidney and urinary tract

**DOI:** 10.1007/s11255-022-03459-z

**Published:** 2023-01-10

**Authors:** Jenny Wei, Amy Showen, Alexandra Bicki, Feng Lin, Charles E. McCulloch, Elaine Ku, Lindsay A. Hampson

**Affiliations:** 1 Department of Medicine, Kaiser Permanente San Francisco, San Francisco, USA; 2 Department of Urology, University of California San Francisco, San Francisco, USA; 3 Division of Pediatric Nephrology, Department of Pediatrics, University of California San Francisco, San Francisco, USA; 4 Department of Epidemiology and Biostatistics, University of California San Francisco, San Francisco, USA; 5 Divisions of Nephrology and Pediatric Nephrology, Department of Epidemiology and Biostatistics, Department of Medicine and Pediatrics, University of California San Francisco, 500 Parnassus Avenue, MBU-E 404, Box 0532, San Francisco, CA 94143-0532 USA; 6 Department of Urology, University of California San Francisco, San Francisco, USA

**Keywords:** Congenital anomalies of the kidney and urinary tract, Renal transplantation, Genetic causes of kidney diesase

## Abstract

**Purpose:**

Although congenital anomalies of the kidney and urinary tract (CAKUT) are among the leading causes of end-stage kidney disease (ESKD) in children and young adults, kidney transplantation access for this population has not been well studied in the US. We compared transplantation access in the US based on whether the etiology of kidney disease was secondary to CAKUT, and additionally by CAKUT subgroups (anatomic vs. inherited causes of CAKUT).

**Methods:**

Using the United States Renal Data System, we conducted a retrospective cohort study of 80,531 children and young adults who started dialysis between 1995 and 2015. We used adjusted Cox models to examine the association between etiology of kidney disease (CAKUT vs. non-CAKUT, anatomic vs. inherited) and receipt of kidney transplantation, and secondarily, receipt of a living vs. deceased donor kidney transplant.

**Results:**

Overall, we found an increased likelihood of kidney transplantation access for participants with CAKUT compared to those without CAKUT (HR 1.23; 95% CI 1.20–1.27). Among the subset of individuals with CAKUT as the attributed cause of ESKD, we found a lower likelihood of kidney transplantation in those with anatomic causes of CAKUT compared to those with inherited causes of CAKUT (adjusted HR 0.85; 0.81–0.90).

**Conclusion:**

There are notable disparities in kidney transplantation rates among CAKUT subgroups. Those with anatomic causes of CAKUT started on dialysis have significantly reduced access to kidney transplantations compared to individuals with inherited causes of CAKUT who were initiated on dialysis. Further studies are needed to understand barriers to transplantation access in this population.

**Supplementary Information:**

The online version contains supplementary material available at 10.1007/s11255-022-03459-z.

## Introduction

Congenital anomalies of the kidney and urinary tract (CAKUT) are one of the main causes of end-stage kidney disease (ESKD) in children and young adults [1–5]. Kidney transplantation is the preferred treatment for individuals with ESKD given that, relative to chronic dialysis, transplantation is associated with better survival, quality of life, and—in pediatric populations—improved growth [6–9]. Furthermore, kidney transplantation is more cost-effective than dialysis as an ESKD treatment modality [10].

While individuals with CAKUT were historically considered to be more complex transplant candidates due to the presence of anatomic urological abnormalities, recent large studies have demonstrated comparable or superior 10-year patient survival in individuals with CAKUT relative to non-CAKUT etiologies of ESKD [5, 11]. Similarly, 10-year graft survival in individuals with CAKUT is at least comparable, if not superior, to graft survival in individuals with non-CAKUT etiologies of ESKD [5, 12, 13]. While earlier outcomes were worse in patients with lower urinary tract dysfunction, more recent data have demonstrated that, with appropriate subspecialty care, transplant recipients with severe lower urinary tract dysfunction have comparable survival and graft function compared with transplant recipients without lower urinary tract dysfunction, despite their higher rates of infectious complications [11, 12, 14, 15].

Although much attention has been focused on patient survival and graft outcomes among individuals with CAKUT and non-CAKUT etiologies of ESKD, less is known about kidney transplantation access in those with CAKUT. One European study which included children and young adults with ESKD demonstrated that individuals with CAKUT initiating dialysis had a similar likelihood of receiving a kidney transplant within 10 years as individuals without CAKUT [11]. However, whether these findings are similar in the US is unknown. If differences in transplantation access by etiology of ESKD are present, there may be important implications for the care of these populations by urologists and nephrologists alike.

The aims of this study are to compare kidney transplantation access among those with CAKUT vs. non-CAKUT etiologies of ESKD, as well as by CAKUT subgroups (which we propose to divide by anatomic vs. inherited causes of CAKUT). We hypothesized that individuals with CAKUT would have better access to transplantation than those without CAKUT due to earlier detection of kidney disease, particularly in the setting of fetal ultrasound imaging advancements. We also hypothesized that transplantation access would be lower in those with inherited causes of CAKUT, especially from living donors, due to the familial nature of the kidney disease (which may preclude living related donors from undergoing donation) compared with anatomic causes of CAKUT.

## Materials and methods

### Study population and data source

We performed a retrospective cohort study of children and young adults < 30 years of age who started chronic dialysis between January 1, 1995 and December 31, 2015 using data from the United States Renal Data System (USRDS), the national ESKD registry [16]. We included individuals up to age 30 years at the time of dialysis initiation, as prior studies have used this upper limit when focusing on young adult populations [17].

Patient demographic characteristics (age at ESKD onset, sex, race), zip code, and date of ESKD onset were abstracted from the Centers for Medicare and Medicaid 2728 (CMS-2728) Medical Evidence Form (submitted at the time of ESKD onset for all US patients) and patients’ file in the USRDS. Zip code was used to determine median household income of patients’ neighborhoods using values from the American Community Survey between 2006 and 2010, which encompasses data from the midpoint of the follow-up period included in our study [18]. Initial ESKD treatment modality (transplant vs. dialysis) was determined at the time of dialysis initiation or kidney transplantation as reported in the patients’ file.

### Primary predictor

We used the PDISP variable to categorize the attributed cause of kidney disease into two overall categories: with CAKUT vs. without CAKUT. We purposefully maintained a broad definition for CAKUT, including any conditions that we thought were congenital or inherited. We further subdivided CAKUT into anatomic-related CAKUT (henceforth referred to as “anatomic causes of CAKUT”) such as posterior urethral valves, vesicoureteral reflux and prune belly syndrome vs. inherited CAKUT disorders (henceforth referred to as “inherited causes of CAKUT”) such as autosomal dominant polycystic kidney disease, tuberous sclerosis, and medullary cystic disease (see Supplemental Table 1), which was guided by European guidelines which recently were published on this division [11]. Diagnoses categorized as non-CAKUT causes of ESKD included glomerulonephritis, diabetes, hypertension, sickle-cell anemia, and others.

### Primary outcomes

The primary outcome was receipt of kidney transplantation, which we ascertained using the USRDS patients’ file. We also examined the hazard of receiving a deceased or living donor transplantation as separate outcomes. Donor source (living vs. deceased donor) was determined from the patients’ file.

### Patient characteristics at dialysis initiation

We ascertained the patients’ demographic characteristics at the start of dialysis in the overall cohort. We also compared characteristics of those who initiated dialysis during childhood (0 to < 18 years of age) vs. those who initiated dialysis as adults (18 to  < 30 years of age) to adhere to the current National Institutes of Health definition of children [19]. We then examined characteristics of individuals based on whether or not their attributed cause of kidney disease was CAKUT (vs. other causes), and then by whether individuals with CAKUT had anatomic or inherited causes of CAKUT.

### Statistical analysis

We used Cox models to examine the association between etiology of ESKD (CAKUT vs. non-CAKUT) and the outcomes of any kidney transplantation, transplantation from a living donor, or transplantation from a deceased donor separately in our primary analyses. These models were adjusted for age at ESKD onset, sex, race/ethnicity, median neighborhood income by patient, zip code, comorbidities at ESKD onset (coronary artery disease, congestive heart failure, stroke, hypertension, diabetes), and dialysis modality. For models with living donor transplantation as the outcome, follow-up was censored at deceased donor transplantation or death. For models with deceased donor transplantation as the outcome, follow-up was censored at living donor transplantation or death. Given that younger age at time of dialysis initiation may associate with greater severity of the CAKUT, we repeated these analyses stratified by age at dialysis initiation (0 to  < 18 years: childhood and 18 to < 30 years: adult). Initially, we excluded patients who received preemptive transplantation, given these individuals may be unique in their access to transplantation when conducting our primary analyses.

We then repeated all analyses among the subgroup of individuals with CAKUT as the attributed cause of their kidney disease in our primary analyses. In these models, we examined whether there were differences in the association between anatomic vs. inherited CAKUT diagnoses and hazard of transplantation (any, living, or deceased donor), adjusted for the same covariates as above.

In additional analyses, we examined the association between CAKUT vs. non-CAKUT and anatomic vs. inherited causes of CAKUT using logistic models among those who received preemptive transplantation given.

All analyses were conducted in SAS 9.0 (SAS Inc, NC). The University of California San Francisco Institutional Review Board considers this study exempt human subjects research.

## Results

### Study population

Our study cohort included 80,531 children and adults less than 30 years of age who either started dialysis or received a preemptive transplantation during our study period. The mean age of the cohort was 22.1 years, 45% were female, 35% were Black, and 40% non-Hispanic White (Table 1). Of those initiated on dialysis, the median follow-up period from time of dialysis initiation until transplantation was 2.53 years. The most common attributed cause of ESKD in the overall cohort was glomerulonephritis (39.3%), and the cohort’s most common co-morbid condition was hypertension (68%) followed by diabetes (18%) (Table 1).

**Table 1:** Baseline characteristics of study cohort

	All data (*N* = 80,531)	Non-CAKUT (*N* = 67,692)	CAKUT (*N* = 12,839)	Anatomic CAKUT (*N* = 8820)	Inherited CAKUT (*N* = 4019)	Preemptive transplants (*N* = 7002)
Age of ESKD service (mean ± SD)	22.1 ± 7.4	23.5 ± 6.3	15.2 ± 9.0	13.8 ± 8.9	18.1 ± 8.6	18.1 ± 8.3
Median income (mean ± SD) (thousands)	49.9 ± 19.7	49.2 ± 19.4	53.4 ± 20.8	52.8 ± 20.5	54.8 ± 21.4	58.4 ± 23.1
Male sex (%)	54.7	52.7	65.0	65.9	63.2	59.5
Race/ethnicity (%)						
Non-Hispanic White	39.9	35.9	61.0	59.0	65.5	71.5
Black	35.4	39.2	15.4	16.6	13.0	10.3
Asian	4.6	4.9	3.0	2.9	3.3	3.5
Hispanic White	18.0	18.0	18.3	19.1	16.4	12.6
Other	2.1	2.1	2.2	2.4	1.8	2.2
Comorbidities at ESKD onset (%)						
CAD	1.1	1.3	0.2	0.2	0.3	0.3
CHF	6.4	7.4	1.3	1.2	1.4	0.7
Stroke	1.3	1.5	0.3	0.3	0.3	0.3
Hypertension	67.5	72.6	40.3	35.4	51.1	47.4
Diabetes	18.3	21.6	1.2	1.1	1.5	8.1
Dialysis modality (%)						
Hemodialysis	72.6	78.0	44.2	41.9	49.0	N/A
Peritoneal dialysis	17.9	15.5	31.0	32.0	28.7	N/A
Unknown	9.5	6.6	24.9	26.0	22.3	N/A
Transplant (%)						
All*	46.2	43.7	52.7	52.1	54.0	100
Living donor	18.9	18.1	23.3	23.2	23.5	69.9
Deceased donor	26.1	25.5	29.2	28.7	30.4	27.8

*CAD* coronary artery disease, *CAKUT* congenital anomalies of the kidney and urinary tract, *CHF* congestive heart failure, *ESKD* end-stage kidney disease, *SD* standard deviation

*All transplant % includes transplants that were designated as Unknown

Within our overall cohort, 12,839 (15.9%) had CAKUT as the attributed cause of ESKD. Compared to those without CAKUT as their cause of ESKD, individuals with CAKUT were more likely to be younger at dialysis initiation, male, non-Hispanic White, and have higher median neighborhood income (Table 1).

Within the CAKUT group, 8820 (68.7%) had anatomic causes of CAKUT, and the remaining had inherited causes of CAKUT (Table 1). Individuals with inherited causes of CAKUT were likely to be older at the time of dialysis initiation compared to individuals with anatomic causes of CAKUT.

Preemptive transplant recipients (*N* = 7002) were younger than non-preemptive transplant recipients (mean age 18.1 ± 8 years vs. 22.1 ± 7.4 years), more likely to be non-Hispanic White (71.5%) and with higher median neighborhood income. Of those with CAKUT as the cause of their ESKD, 30.0% (*N* = 3857) received a preemptive transplant. Of individuals with anatomic causes of CAKUT, 25.6% (*N* = 2260) received a preemptive transplant while 22.0% (*N* = 885) of those with inherited causes of CAKUT received a preemptive transplant.

### Transplantation access in CAKUT vs. non-CAKUT ESKD

**Fig. 1:**
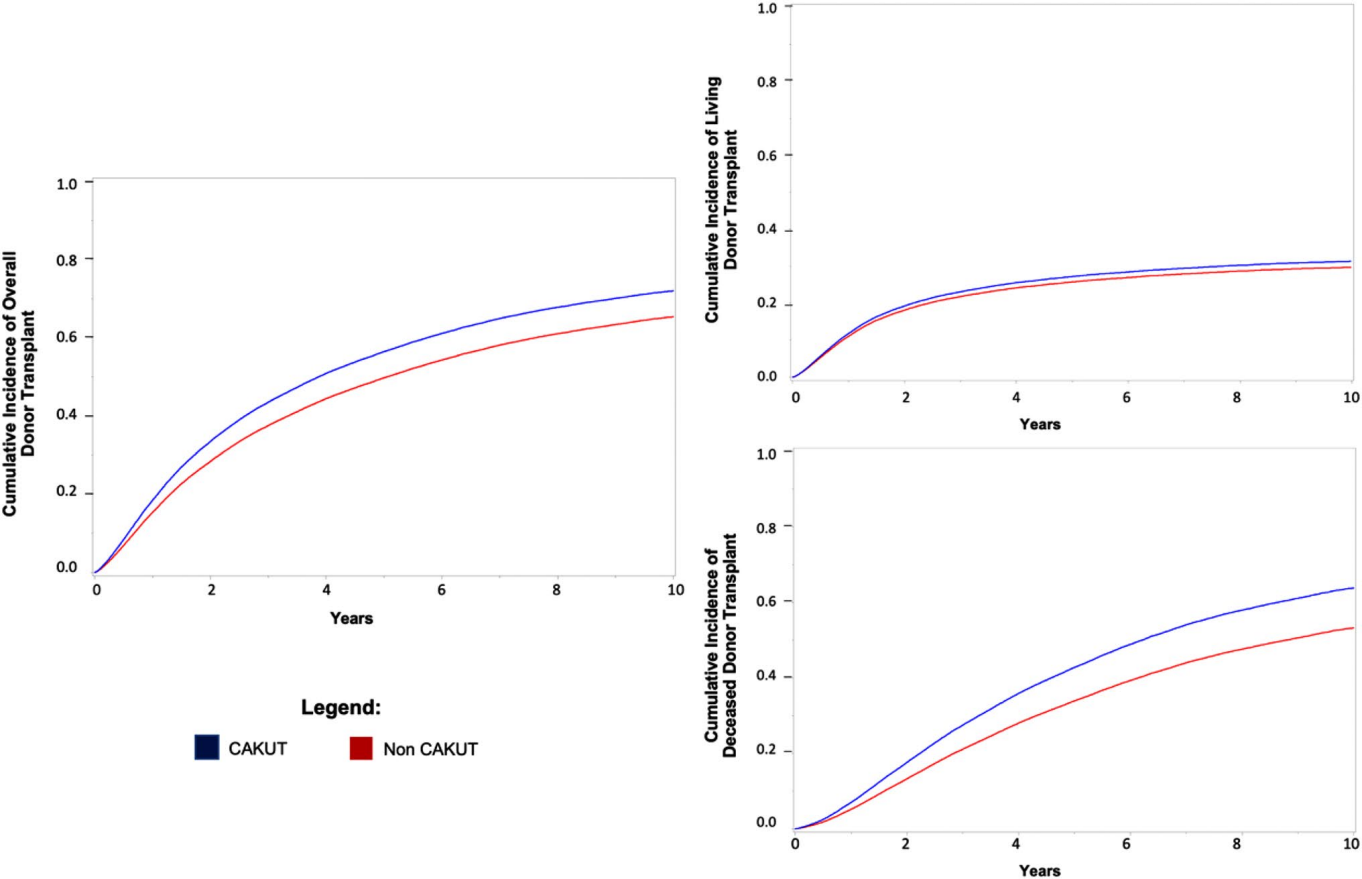
Time to overall, living or deceased donor transplant comparing CAKUT to non-CAKUT groups

Within our overall cohort, 29,575 (43.7%) of individuals without CAKUT as their attributed cause of ESKD received a kidney transplant (median time from dialysis initiation to transplant was 2.71 years) compared with 7,061 (55.0%) individuals with CAKUT (median time from dialysis initiation to transplant was 1.62 years). Among those with CAKUT, 4592 (52.1%) of those with anatomic causes of CAKUT received a transplant (median time from dialysis initiation to transplant was 1.65 years [IQR 0.70–3.66]) compared to 2169 (54.0%) individuals with inherited causes of CAKUT (median time from dialysis initiation to transplant was 1.54 years [IQR 0.68–3.49]). We found a higher hazard of both living donor transplantation (HR 1.07; 95% CI 1.03–1.12, Fig. 1) and deceased donor transplantation (HR 1.37; 95% CI 1.32–1.43, Fig. 1) in those with CAKUT compared to those without CAKUT as causes of their ESKD.

### Transplantation access in CAKUT ESKD, by age at dialysis initiation

In subgroup analysis stratified by age of dialysis initiation, we found that patients with CAKUT who started dialysis in childhood were more likely to receive both living and deceased donor transplantation compared to those who started dialysis in childhood without CAKUT (Table 2). This trend was similar for adults who started dialysis as well, where we found that adults with CAKUT were more likely to receive both living and deceased donor transplants compared to adults without CAKUT (Table 2).

**Table 2:** Any, living, or deceased donor transplant stratified by age of dialysis initiation

	Hazard ratio (95% CI HR)	
	CAKUT vs. non-CAKUT (reference group)	Anatomic CAKUT vs. inherited CAKUT (reference group)
Children (< 18 years)		
Living donor transplant	1.09 (1.02–1.16)	0.91 (0.81–1.02)
Deceased donor transplant	1.28 (1.21–1.35)	0.76 (0.69–0.83)
Adults (18– < 30 years)		
Living donor transplant	1.07 (1.01–1.14)	0.93 (0.83–1.04)
Deceased donor transplant	1.32 (1.24–1.40)	0.70 (0.63–0.79)

Model above is adjusted for age at ESKD onset, sex, race/ethnicity, median neighborhood income, comorbidities at ESKD onset and initial dialysis modality

### Transplantation access in CAKUT ESKD, by attributed cause (anatomic vs. inherited)

**Fig. 2:**
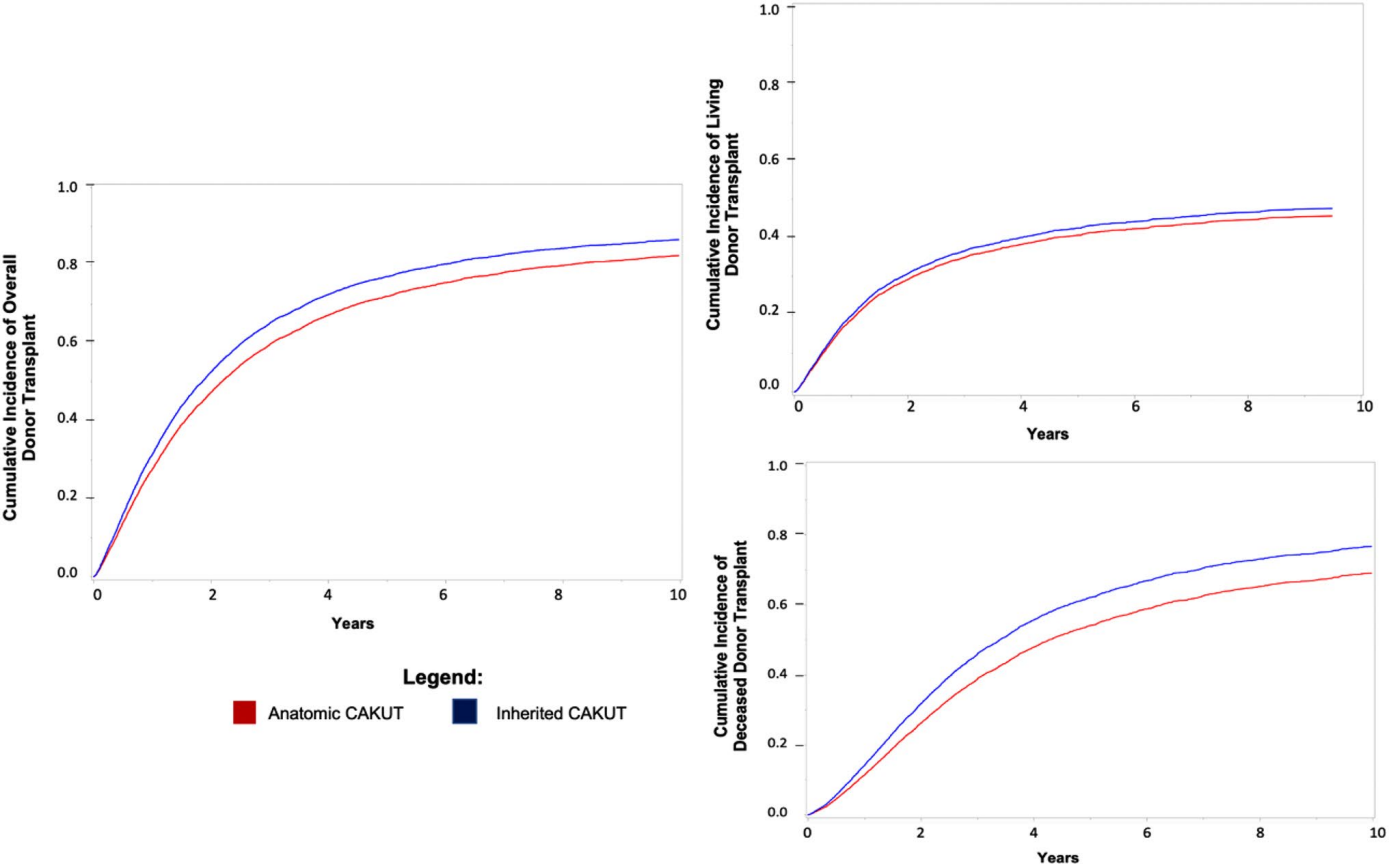
Time to overall, living or deceased donor transplant comparing anatomic causes vs. inherited causes of CAKUT subgroups

Among the subset of individuals with CAKUT as the attributed cause of ESKD who started dialysis, we found a statistically lower likelihood of transplantation for those with anatomic vs. inherited causes of CAKUT (HR 0.85; 95% CI 0.81–0.90, Fig. 2). Individuals with anatomic causes of CAKUT had a significantly lower hazard of receiving a deceased donor transplant (HR 0.79; 95% 0.74–0.85) than those with inherited causes of CAKUT, while the rates of living donor transplantation were similar in both groups (HR 0.94; 95% CI 0.87–1.02). This trend was observed in both children and adults.

### Preemptive transplantation access

The odds of preemptive transplantation were 2.20 times higher (95% CI 1.87–2.58) in those with CAKUT vs. those with non-CAKUT causes of their ESKD and the odds of preemptive transplantation in those with anatomic causes of CAKUT were similar to those with inherited causes of CAKUT (HR 1.07; 95% CI 0.78–1.47).

## Discussion

To our knowledge, this study is the first to examine kidney transplantation access in children and adults with CAKUT in the US. While a previous European study found that individuals with CAKUT starting dialysis had a similar likelihood of receiving a kidney transplant within 10 years of starting dialysis compared with individuals with other causes of ESKD, we found that individuals with CAKUT were more likely to receive a deceased and living donor transplant than individuals without CAKUT [11]. Given that many causes of CAKUT are detected prenatally, the higher rates of transplantation for individuals with CAKUT compared to individuals without CAKUT may be due to earlier recognition of disease and preparation for transplantation, which is supported by the higher odds of preemptive transplantation overall in those with CAKUT. Earlier recognition of disease may also lead to earlier waitlisting and ensure early accrual of wait time, which may contribute to better access to deceased donor transplantation.

In subgroup analysis of those with CAKUT, we found that individuals with anatomic causes of CAKUT initiated on dialysis were less likely to receive deceased kidney donor transplantation compared to those with inherited causes of CAKUT who were initiated on dialysis. However, those with anatomic causes of CAKUT had comparable levels of living donor transplantation compared to those with inherited causes of CAKUT. We had hypothesized that those with inherited causes of CAKUT would be less likely to receive a living donor transplant compared to those with anatomic causes of CAKUT given that those with inherited causes of ESKD may have fewer eligible related living donors, but this was not what we observed. Since anatomic causes of ESKD are frequently diagnosed early in an individual’s life, the disparity in transplantation access for this population compared to those with inherited causes of CAKUT is concerning. Many anatomic causes of CAKUT can predispose individuals to lower urinary tract dysfunction, which carries a high risk of post-transplant bladder dysfunction [20]. Thorough assessment of transplant candidacy in those with lower urinary tract dysfunction improves the likelihood of graft success. This involves voiding cystourethrogram and urodynamic studies to evaluate capacity, compliance, post-void residual urine, bladder function and presence of lower urinary tract reflux [21]. We, therefore, speculate that these individuals may have required additional medical or surgical interventions such as nephrectomy and bladder augmentation to optimize their lower urinary tract and reduce events of urological complications and urinary tract infections [13, 22]. It is also possible that there may be concerns surrounding compliance with catheterization and low body weight requiring growth hormone and significant nutritional support that contributes to delayed transplantation in this group [23]. These additional obstacles could explain the lower likelihood of deceased kidney transplantation in those with anatomic vs. inherited causes of their ESKD.

Interestingly, among CAKUT subgroups, the findings regarding access to preemptive transplantation vs. non-preemptive transplant are not congruent. Those with anatomic CAKUT on dialysis were less likely to receive non-preemptive donor transplantations compared to those with inherited causes of CAKUT, whereas those with anatomic CAKUT had similar rates of receiving preemptive transplantation compared to those with inherited causes of CAKUT. We speculate that this may be due to differences in the number of underlying issues that needed resolution prior to transplantation in the dialysis group with anatomic CAKUT vs. the preemptive transplantation group with anatomic CAKUT who were transplant ready. Although the median difference in time to transplant between those with anatomic causes compared with inherited causes was only approximately 2 months, prior studies have shown that even a short duration of exposure to dialysis is associated with worse outcomes in individuals with ESKD [24, 25].

Importantly, we highlight that the definition of CAKUT has varied significantly in the literature across studies [4, 5, 26, 27]. For example, while some studies do not consider polycystic kidney diseases as CAKUT, other studies categorize cystic kidney diseases as part of CAKUT [5, 26, 28, 29]. Furthermore, the subdivision of CAKUT itself also lacks consensus due to the group’s heterogeneity. Melo et al. divide CAKUT into four major subgroups: urinary tract dilatation, renal cystic diseases, renal agenesis and miscellaneous, whereas Sanna-Cherchi et al. describe six major phenotypes of CAKUT: solitary kidney, bilateral hypodysplasia, unilateral hypodysplasia, multicystic kidney, horseshoe kidney, and posterior urethral valves [27, 29]. Some studies describe CAKUT as urological causes of ESKD with a focus on the subcategory of lower urinary tract dysfunction due to the need for extensive preparation of the lower urinary tract for safe transplantation [15, 27]. Others have excluded inherited conditions altogether from their CAKUT study group. [28] Due to the absence of standardized CAKUT definitions, studies that examine these conditions and clinical outcomes may be difficult to compare. We have provided one approach to the division of CAKUT to refine our understanding of access to transplantation here and shown the heterogeneity within those with CAKUT and the importance of examining sub-phenotypes and their association with outcomes [13, 32]. We acknowledge, however, that there may be other approaches to define these subtypes. Given the current lack of uniformity in categorization, a formal and systematic definition of CAKUT and its subgroups should be developed for better guidance on prognosis, risk assessment, and follow-up for both nephrologists and urologists.

Our study has several strengths. It is one of the first studies to examine transplantation access in individuals with CAKUT from infancy to age 30 and is unique in the sub-phenotyping of CAKUT that we provide when examining outcomes of interest. Our study also includes a large cohort of individuals with CAKUT who started dialysis in the US across two decades. We note several limitations to our study. First, there is the potential for misclassification of the cause of ESKD based on administrative data; for example, some individuals may have multiple diagnoses spanning across anatomic and inherited causes of CAKUT. Given the heterogeneity of CAKUT, categorization of diagnoses may differ across specialties and practitioners. In addition, as in all observational studies, residual confounding may be present. Finally, we do not have granular data on the reasons for lower access of those with anatomic causes to transplantation, and this will be an important future area of study.

In conclusion, our study indicates that there are disparities in transplantation access for children and adults with ESKD secondary to CAKUT. While individuals with CAKUT are more likely to receive preemptive, living and deceased kidney transplants compared to those without CAKUT etiologies of ESKD, those with anatomic causes of CAKUT who have started dialysis are less likely to receive deceased transplants compared to those with inherited causes of CAKUT who have started dialysis. Further research is needed to determine the barriers hindering transplant access among individuals with anatomic causes of CAKUT in an effort to improve life expectancy and reduce morbidity within this patient population.

## Supplementary Information

Below is the link to the electronic supplementary material.

Supplementary file1 (DOCX 14 kb)

## Data Availability

These data were derived from the following resource available in the public domain: United States Renal Data System (https://usrds-adr.niddk.nih.gov/).

## Abbreviations

CAKUT: Congenital anomalies of the kidney and urinary tract
CI: Confidence interval
ESKD: End-stage kidney disease
HR: Hazard ratio
Non-CAKUT: Non-congenital anomalies of the kidney and urinary tract
SD: Standard deviation
US: United States
USRDS: United States renal data system
