# Supplementary material for: Kidney transplant access for children and young adults with congenital anomalies of the kidney and urinary tract
Source: Int Urol Nephrol. 2023 Jan 10;55(6):1531–8. doi: 10.1007/s11255-022-03459-z (PMC10185613; doi:10.1007/s11255-022-03459-z)
Supplement: Supplementary file 1 — Supplementary file1 (DOCX 14 kb) [file 11255_2022_3459_MOESM1_ESM.docx]

**Supplemental Table 1:  Classification of CAKUT by Diagnoses**

| **DIAGNOSIS** | **CAKUT SUBCATEGORY** |
| --- | --- |
| UNILATERAL SMALL KIDNEY | CAKUT - Anatomic |
| BILATERAL SMALL KIDNEY | CAKUT - Anatomic |
| SMALL KIDNEY, UNSPECIFIED | CAKUT - Anatomic |
| SMALL KIDNEY OF UNKNOWN CAUSE | CAKUT - Anatomic |
| CHRONIC PYELONEPHRITIS, REFLUX NEPHROPATHY | CAKUT - Anatomic |
| OBSTRUCTIVE KIDNEY / OBSTRUCTIVE NEPHROPATHY | CAKUT - Anatomic |
| RENAL HYPOPLASIA, DYSPLASIA, OLIGONEPHRONIA | CAKUT - Anatomic |
| HYPOPLASIA KIDNEY DISEASE / SOLITARY KIDNEY | CAKUT - Anatomic |
| CONGENITAL OBSTRUCTION OF URETEROPELVIC JUNCTION | CAKUT - Anatomic |
| OTHER CONGENITAL OBSTRUCTIVE UROPATHY | CAKUT - Anatomic |
| CONGENITAL OBSTRUCTIVE UROPATHY | CAKUT - Anatomic |
| OBSTRUCTIVE DEFECTS OF RENAL PELVIS AND URETER | CAKUT - Anatomic |
| CALCULI RENAL, CONGENITAL / OBSTRUCTIVE UROPATHY, CONGENITAL | CAKUT - Anatomic |
| DYSPLASIA (ANOMALY) OF KIDNEY | CAKUT - Anatomic |
| CONGENITAL ANOMALIES OF URINARY SYSTEM / RENAL AGENESIS AND DYSGENESIS | CAKUT - Anatomic |
| VESICOURETERAL-REFLUX, UNSPECIFIED | CAKUT - Anatomic |
| OTHER OBSTRUCTIVE AND REFLUX UROPATHY | CAKUT - Anatomic |
| OBSTRUCTIVE UROPATHY/VESICOURETERAL REFLUX, UNSPECIFIED | CAKUT - Anatomic |
| NEUROMUSCULAR DYSFUNCTION OF BLADDER, UNSPECIFIED | CAKUT - Anatomic |
| HERMAPHRODITISM, NOT ELSEWHERE CLASSIFIED | CAKUT - Anatomic |
| RENAL AGENESIS, UNSPECIFIED | CAKUT - Anatomic |
| RENAL DYSPLASIA | CAKUT - Anatomic |
| CONGENITAL OCCLUSION OF URETEROPELVIC JUNCTION | CAKUT - Anatomic |
| CONGENITAL OCCLUSION OF URETEROVESICAL ORIFICE | CAKUT - Anatomic |
| OTHER SPECIFIED CONGENITAL MALFORMATIONS OF KIDNEY | CAKUT - Anatomic |
| CONGENITAL POSTERIOR URETHRAL VALVES | CAKUT - Anatomic |
| PRUNE BELLY SYNDROME | CAKUT - Anatomic |
| OTH CONGEN MALFORM SYNDROMES DUE TO KNOWN EXOGENOUS CAUSES | CAKUT - Anatomic |
| CYSTIC KIDNEY DISEASE / MEDULLARY CYSTIC, MULTICYSTIC, POLYCYSTIC KIDNEY DISEASE | CAKUT - Anatomic |
| CYSTINOSIS | CAKUT - Inherited |
| CYSTINOSIS, MALIGNANT | CAKUT - Inherited |
| PRIMARY OXALOSIS | CAKUT - Inherited |
| OXALATE NEPHROPATHY / OXALOSIS | CAKUT - Inherited |
| FABRY'S DISEASE | CAKUT - Inherited |
| POLYCYSTIC KIDNEYS, ADULT TYPE (DOMINANT) | CAKUT - Inherited |
| POLYCYSTIC, INFANTILE (RECESSIVE) | CAKUT - Inherited |
| MEDULLARY CYSTIC DISEASE, INCLUDING NEPHRONOPHTHISIS | CAKUT - Inherited |
| TUBEROUS SCLEROSIS | CAKUT - Inherited |
| HEREDITARY NEPHRITIS, ALPORT'S SYNDROME | CAKUT - Inherited |
| HEREDITARY/FAMILIAL NEPHROPATHY | CAKUT - Inherited |
| ALPORT'S SYNDROME | CAKUT - Inherited |
| CYSTINOSIS | CAKUT - Inherited |
| HYPEROXALURIA | CAKUT - Inherited |
| FABRY (-ANDERSON) DISEASE | CAKUT - Inherited |
| HEREDITARY NEPHROPATHY, NEC W MINOR GLOMERULAR ABNORMALITY | CAKUT - Inherited |
| HEREDITARY NEPHROPATHY, NEC W OTH MORPHOLOGIC LESIONS | CAKUT - Inherited |
| OTHER POLYCYSTIC KIDNEY, INFANTILE TYPE | CAKUT - Inherited |
| POLYCYSTIC KIDNEY, ADULT TYPE | CAKUT - Inherited |
| MEDULLARY CYSTIC KIDNEY | CAKUT - Inherited |
| OTHER CYSTIC KIDNEY DISEASES | CAKUT - Inherited |
| TUBEROUS SCLEROSIS | CAKUT - Inherited |
| ALPORT'S SYNDROME | CAKUT - Inherited |
